# Development of a Candidate ^11^C‑Labeled Selective Phosphodiesterase 1 Radioligand for Positron Emission Tomography

**DOI:** 10.1021/acsomega.4c03214

**Published:** 2024-10-21

**Authors:** Jian Rong, Tomoteru Yamasaki, Jiahui Chen, Katsushi Kumata, Chunyu Zhao, Masayuki Fujinaga, Kuan Hu, Wakana Mori, Yiding Zhang, Lin Xie, Ahmad F. Chaudhary, Xin Zhou, Wei Zhang, Yabiao Gao, Kuo Zhang, Jimmy S. Patel, Zhendong Song, Thomas L. Collier, Hongjie Yuan, Chongzhao Ran, Achi Haider, Yinlong Li, Ming-Rong Zhang, Steven Liang

**Affiliations:** † Department of Radiology and Imaging Sciences, 1371 Emory University, Atlanta, Georgia 30322, United States; ‡ Division of Nuclear Medicine and Molecular Imaging, Massachusetts General Hospital, and Department of Radiology, Harvard Medical School, Boston, Massachusetts 02114, United States; § Department of Advanced Nuclear Medicine Sciences, Institute for Quantum Medical Science, National Institutes for Quantum Science and Technology, Chiba 263-8555, Japan; ∥ Department of Radiation Oncology, Winship Cancer Institute of Emory University, Atlanta, Georgia 30322, United States; ⊥ Department of Pharmacology and Chemical Biology, 12239 Emory University School of Medicine, Atlanta, Georgia 30322, United States; # Athinoula A. Martinos Center for Biomedical Imaging, Department of Radiology, Massachusetts General Hospital and Harvard Medical School, Boston, Massachusetts 02114, United States

## Abstract

Phosphodiesterases (PDEs) constitute a superfamily of phosphohydrolytic enzymes that regulate intracellular second messenger signaling by hydrolyzing cyclic adenosine monophosphate and cyclic guanosine monophosphate. Among the 11 subfamilies of PDEs, phosphodiesterase 1 (PDE1) stands out due to its broad implications in central and peripheral pathologies. There are three subtypes of PDE1: PDE1A, PDE1B, and PDE1C. While PDE1A and PDE1C are distributed in both the brain and peripheral organs, PDE1B is predominantly expressed in the brain, rendering it an attractive drug target for neurological and psychological disorders. Despite continuous efforts dedicated to the development of novel PDE1 inhibitors, a suitable PDE1 radioligand for human use is currently lacking. In this study, we present the identification and preclinical evaluation of [^11^C]­PF-04822163, a selective radioligand candidate for imaging PDE1 with positron emission tomography. PF-04822163 exhibits excellent potency toward PDE1 and demonstrates great target selectivity over other PDEs. Then, PF-04822163 was labeled with carbon-11 (half-life, 20 min) in favorable radiochemical yields (25 ± 10%, decay-corrected) and high molar activities (106–194 GBq/μmol). Further, *in vitro* and *in vivo* evaluations in rodents suggested that [^11^C]­PF-04822163 displayed good brain penetration and a rapid washout. Despite these promising performance characteristics of [^11^C]­PF-04822163, only marginal specific binding was observed *in vivo*. Further optimization of the scaffold is warranted to obtain favorable pharmacological and ADME properties.

## Graphical abstract

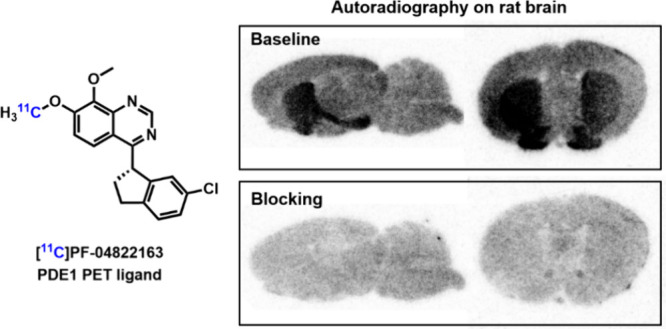

## Introduction

Phosphodiesterases (PDEs) are phosphohydrolytic enzymes responsible for the degradation of cyclic adenosine monophosphate (cAMP) and cyclic guanosine monophosphate (cGMP), which are second messengers that regulate various intracellular signaling processes. To date, PDEs have been categorized into 11 subfamilies, designated PDEs 1 through 11. Among these, PDEs 4, 7, and 8 target cAMP, and PDEs 5, 6, and 9 target cGMP, while PDEs 1, 2, 3, 10, and 11 target both cAMP and cGMP.^1,2^ The PDE1 subfamily has three members, PDE1A, PDE1B, and PDE1C, which exhibit varying affinities to cAMP and cGMP and display distinct tissue distributions. PDE1A and PDE1B demonstrate a higher affinity for cGMP over cAMP, while PDE1C exhibits similar affinities for both cGMP and cAMP.^2,3^ PDE1A is mainly expressed in the kidney, thyroid gland, and brain, especially in the cortex and hippocampus. PDE1C is mainly found in the heart and brain.^4,5^ PDE1B is primarily expressed in the brain, particularly in regions such as the striatum, substantia nigra, nucleus accumbens, and olfactory tubercle.^6^ Given its predominant expression in brain regions associated with learning and memory, PDE1B has emerged as a promising target for drug development aimed at the treatment of neurological and neurodegenerative disorders such as Alzheimer’s disease.^7^

Significant efforts have been directed toward the development of novel and selective PDE1 inhibitors (Figure 1 and Figure S1 in the Supporting Information).^8^ Initially, inhibitors were found to exhibit moderate inhibitory potencies against PDE1 while also affecting other targets. Vinpocetine is one such PDE1 inhibitor (PDE1 IC_50_ = 30 μM),^9^ and it also acts as an inhibitor of voltage-dependent Na^+^ channels^10,11^ as well as IκB kinases.^12^ In recent years, an increasing number of PDE1 selective inhibitors have been identified.^8^ An exemplary PDE1 selective inhibitor, ITI-214, demonstrated subnanomolar binding affinity toward PDE1 and remarkable target selectivity (PDE1 IC_50_ = 0.058 nM, >1000-fold over other PDEs).^13^ Along this line, ITI-214 has shown efficacy in enhancing learning and memory in rodents.^13,14^ Currently, ITI-214 has completed three phase I studies, including assessments of central nervous system (CNS) engagement in healthy volunteers (NCT03489772) and evaluations in patients with Parkinson’s disease (PD) (NCT03257046) and heart failure (NCT03387215). Another notable development is the quinazoline-based inhibitor,^15^ PF-04822163, which exhibits potent PDE1 inhibition and remarkable target selectivity (PDE1B IC_50_ = 2.4 nM, >100-fold selectivity over other PDEs). Additionally, other selective PDE1 inhibitors such as DSR-141562,^16^ DNS-0056,^17^ Lu AF58027,^18,19^ SCH-51866,^20,21^ PF-04471141,^15^ and PF-04827736 ^22^ have been developed.

**Figure 1:**
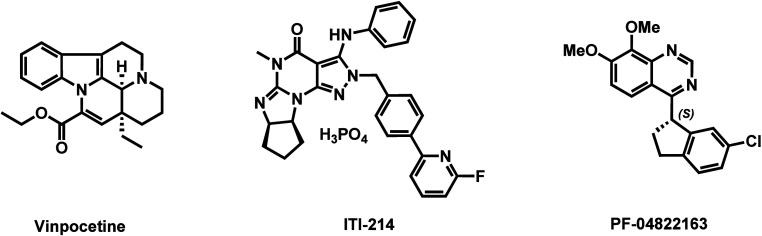
Representative PDE1 inhibitors.

Positron emission tomography (PET) is a noninvasive medical imaging technique that offers quantitative functional insights into biological processes at the molecular level by detecting signals from PET tracers *in vivo*.^23,24^ PET imaging of PDE1 could enable visualization of PDE1 *in vivo* and assessment of PDE1 levels under both healthy and diseased conditions, thereby facilitating the development of therapeutic PDE1 inhibitors. However, despite the progress made in the development of PDE1 inhibitors, there remains a critical gap: the absence of a clinically available PDE1 PET ligand. Only a handful of examples were reported in meeting abstracts and patents, which are primarily at the radiochemistry and preclinical evaluation stages.^8^ These underscore an urgent and unmet need to develop specific probes for imaging PDE1. In collaboration with the Pfizer drug discovery team, we report on [^11^C]­PF-04822163 as a PDE1 PET ligand, including the radiosynthesis of enantiomerically pure [^11^C]­PF-04822163 and the subsequent *in vitro* and *in vivo* evaluations.

## Results and Discussion

As shown in Figure 2, PF-04822163 demonstrated exceptional inhibitory activity against PDE1 with high potency of IC_50_ values of 2.0, 2.4, and 7.0 nM for PDE1A, PDE1B, and PDE1C, respectively (Figure 2b), together with great target selectivity, exhibiting 126-, 105-, and 36-fold selectivity over other PDEs for PDE1A, PDE1B, and PDE1C, respectively (Figure 2b,c).^15^ The topological polar surface area (tPSA= 44.24) was predicted by Chemicalize,^25^ an online platform for chemical calculations, and log *D* (2.53) was measured with [^11^C]­PF-04822163 by the “shake flask method”, suggesting an appropriate lipophilicity property for PF-04822163. Permeability across the blood–brain barrier (BBB) of PF-04822163 was predicted with ACD/Percepta, and the result (log BB = 0.33) suggested a high likelihood (log BB > −1) of BBB permeability.^26^ Additionally, the central nervous system multiparameter optimization (CNS MPO) algorithm was used to predict the BBB permeability, and the CNS MPO score (4.1, using a scale of 0–6) indicated that PF-04822163 has a high probability of BBB permeability (MPO score ≥4).^27^ Furthermore, we investigated the off-target binding of PF-04822163 *in vitro* against 65 major CNS targets, including ion channels, transporters, and GPCR enzymes (Figure S2 in the Supporting Information, supported by the NIMH Psychoactive Drug Screening Program (PDSP)). There was no significant off-target binding (>50% inhibition) for PF-04822163 at a 10 μM testing concentration, except for 5-HT2B (*K*_i_ = 262 nM) and 5-HT6 (*K*_i_ = 4858 nM).

**Figure 2:**
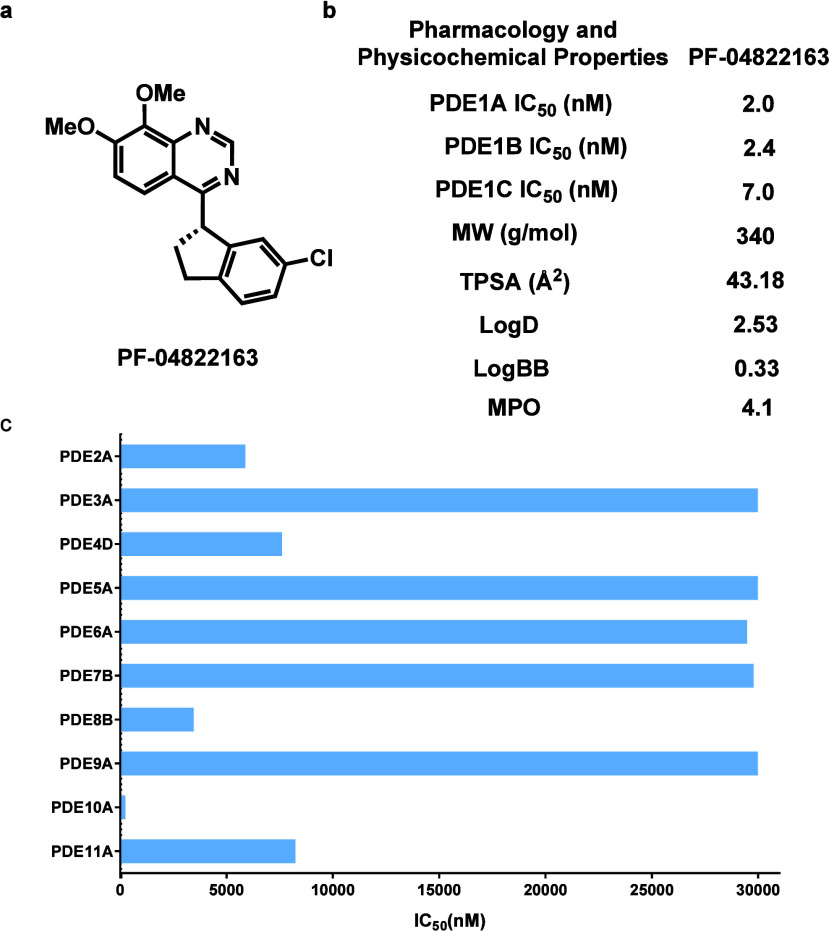
Pharmacological profile of PF-04822163. (a) Chemical structure of PF-04822163, (b) representative pharmacological and physicochemical properties of PF-04822163, and (c) potency of PF-04822163 toward other PDEs (IC_50_ of 252 nM for PDE10A).

With [^11^C]­PF-04822163 as the PDE1 PET ligand of interest, we conducted an efficient synthesis of the phenolic precursor for ^11^C-labeling of PF-04822163. Indeed, the synthesis of precursor **10** of PF-04822163 was accomplished as shown in Scheme 1. Nitration of compound **1** with fuming nitric acid, followed by the oxidation of compound **2** with potassium permanganate, afforded compound **3** in 72% yield over two steps. The reduction of compound **3** was achieved quantitatively using palladium on carbon and hydrogen gas, followed by the cyclization reaction of compound **4** with formamidine acetate, affording key intermediate **5** in 17% yield. In the next step, compound **7** was generated via acetylation of intermediate **5** and a chlorination reaction of compound **6** in 47% yield over two steps. A nucleophilic substitution reaction between compounds **7** and methyl 6-chloro-2,3-dihydro-1*H*-indene-1-carboxylate and hydrolysis and decarboxylation of compound **8** gave racemic precursor **9** in 61% yield over three steps. Finally, the chiral separation of racemic precursor **9** generated compound **10** as the precursor of PF-04822163. The absolute configuration of the precursor (**10**) was identified according to that of PF-04822163. PF-04822163 was prepared from the methylation of **10**, and PF-04822163 was identified as an *S*-enantiomer by optical rotation data consistent with a previous report.^15^

**Scheme 1:**
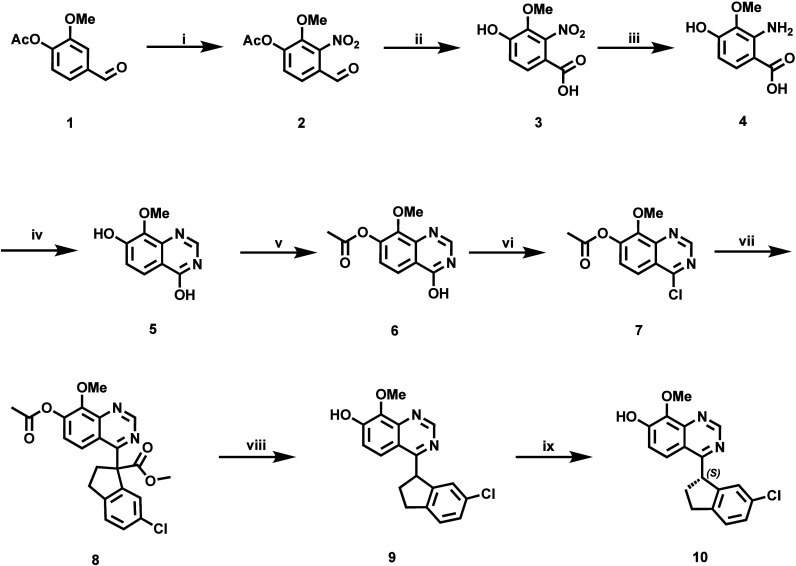
Synthesis of the Labeling Precursor **10** of [^11^C]­PF-04822163^a^

a (i) Fuming HNO_3_, 0–5 °C, 1 h, 73% yield; (ii) KMnO_4_, acetone, 0 °C to room temperature, 12 h, 98% yield; (iii) Pd/C, H_2_, MeOH, room temperature, 12 h, 100% yield; (iv) formamidine acetate, 2-methoxyethanol, reflux, 18 h, 17% yield; (v) Ac_2_O, pyridine, 100 °C, 4 h, 85% yield; (vi) Vilsmeier reagent, CH_2_Cl_2_, room temperature, 2 h, 55% yield; (vii) methyl 6-chloro-2,3-dihydro-1*H*-indene-1-carboxylate, potassium bis­(trimethylsilyl)­amide, tetrahydrofuran, 0 °C, 30 min; (viii) NaOH (aq. 1 N), MeCN, microwave, 15 min, 130 °C, 61% yield over two steps; (ix) chiral separation, 47% yield.

PF-04822163 was then radiolabeled with carbon-11 from the enantiopure phenolic precursor **10** and further evaluated as a PDE1 PET radioligand. We chose PF-04822163 due to its favorable pharmacological and physicochemical properties (PDE1B IC_50_: 2.4 and 458 nM for PF-04822163 and its *R*-enantiomer, respectively).^15^ Parallel efforts have been made by others in the ^11^C-labeling of PF-04780905 (a racemic version of PF-04822163) and reported in meeting abstracts.^28,29^ PF-04780905 was ^11^C-labeled in 10% decay-corrected RCY with [^11^C]­CH_3_I and Cs_2_CO_3_ as the base in DMSO at 80 °C for 5 min. The radiosynthesis of [^11^C]­PF-04822163 involved ^11^C-methylation of the corresponding phenolic precursor **10** with [^11^C]­CH_3_I and NaOH as the base in DMF at 30 °C for 5 min (Scheme 2). Starting from [^11^C]­CO_2_, [^11^C]­PF-04822163 was generated in 25 ± 10% radiochemical yield (decay-corrected, *n* = 5) with high molar activities of 106–194 GBq/μmol (*n* = 5) at the end of synthesis. The radiochemical purity of [^11^C]­PF-04822163 was greater than 99%, and the enantiomeric purity of [^11^C]­PF-04822163 was 98% (96% ee).

**Scheme 2:**
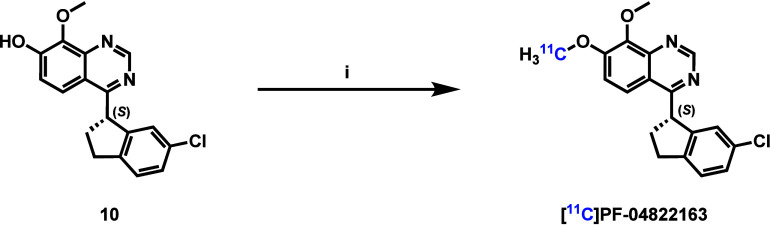
Radiosynthesis of [^11^C]­PF-04822163^a^

a Labeling conditions: (i) [^11^C]­CH_3_I, NaOH, DMF, 30 °C, 5 min, 25 ± 10% radiochemical yield (decay-corrected) with a molar activity of 106.1–194.1 GBq/μmol (*n* = 5). DMF = dimethylformamide.

With the ligand [^11^C]­PF-04822163 in hand, *in vitro* autoradiography studies were conducted on Sprague–Dawley (SD) rat brain sections to validate its binding specificity toward PDE1. As shown in Figure 3a, [^11^C]­PF-04822163 demonstrated a heterogeneous brain distribution in sagittal rat brain sections. High radioactivity levels were observed in the striatum and substantia nigra followed by the cerebral cortex, hippocampus, and thalamus, while lower radioactivity levels were found in the cerebellum and pons (Figure 3a,b). In the autoradiography studies on the coronal brain sections, high radioactivity levels were found in the caudate putamen, nucleus accumbens, and olfactory tubercle (Figure 3a,c). In the blocking studies, pretreatment with unlabeled PF-04822163 or PF-04827736 (a PDE1B inhibitor with an IC_50_ value of 9.1 nM) both led to a significant decrease in radioactivity signal in PDE1B-rich regions (a decrease of 47–71% for PF-04822163 and a decrease of 24–51% for PF-04827736), indicating favorable *in vitro* binding specificity of [^11^C]­PF-04822163 toward PDE1B (Figure 3a–c).

**Figure 3:**
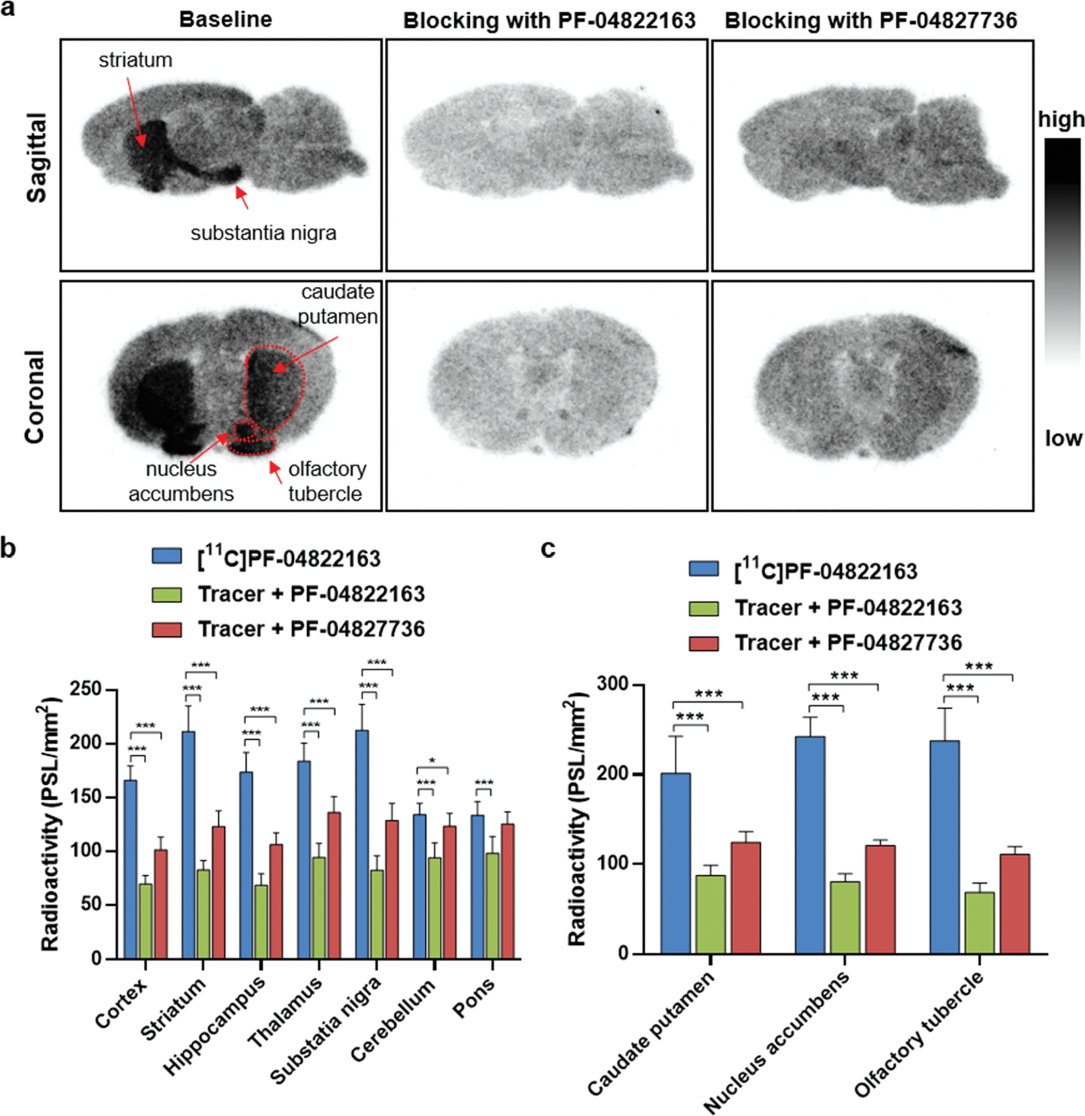
*In vitro* autoradiography studies of [^11^C]­PF-04822163 on rat brain sections. (a) Representative images of autoradiography studies with [^11^C]­PF-04822163 under baseline and blocking conditions (PF-04822163 or PF-04827736, 10 μM), (b) quantification of autoradiography studies of [^11^C]­PF-04822163 on sagittal rat brain sections, and (c) quantification of autoradiography studies of [^11^C]­PF-04822163 on coronal rat brain sections. All data are means ± SD, *n* ≥ 8. Statistical analysis was conducted with Student’s two-tailed *t* tests, and asterisks indicated the statistical significance as follows: *p < 0.05, **p ≤ 0.01, and ***p ≤ 0.001.

Encouraged by the results of *in vitro* evaluation by autoradiography studies with the ligand [^11^C]­PF-04822163, dynamic PET imaging studies were conducted with [^11^C]­PF-04822163 on SD rats. As shown in Figure 4, [^11^C]­PF-04822163 efficiently penetrated the BBB with the highest radioactivity accumulation observed in the striatum (SUV_max_ = 2.0 at 3 min post-tracer administration) followed by the hippocampus, cortex, cerebellum, and the lowest radioactivity accumulation in the pons (Figure 4a,b). Notably, the initial uptake of [^11^C]­PF-04822163 in different regions of the brain in the baseline study was consistent with the results of autoradiography studies (Figure 3). Additionally, an evident washout was observed after initial high uptakes in all brain regions. To assess the *in vivo* binding specificity of [^11^C]­PF-04822163 to PDE1, we carried out blocking studies with the pretreatment of unlabeled PF-04822163 (1 mg/kg, 10 min before the administration of [^11^C]­PF-04822163). Pretreatment with unlabeled PF-04822163 reduced the radioactivity level in all brain regions, and the corresponding areas under the curve (AUC) revealed a marginal reduction of 7–12% in uptake in different brain regions (Figure 4c). These findings indicate that further optimization of the pharmacological and ADME properties is warranted to obtain a suitable probe for *in vivo* imaging of PDE1.

**Figure 4:**
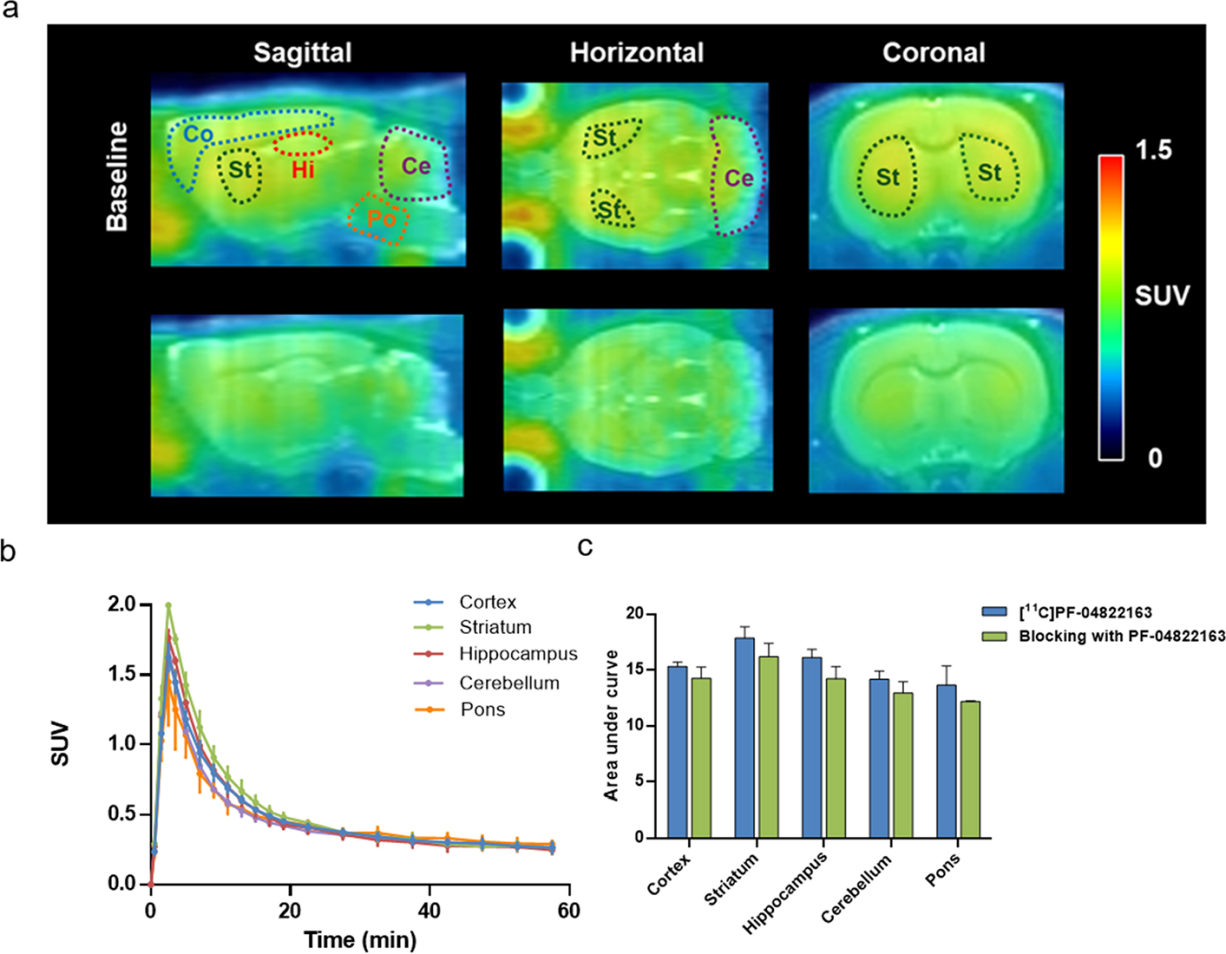
PET studies of [^11^C]­PF-04822163 in rat brains. (a) Representative summed PET images (0–20 min) of [^11^C]­PF-04822163 in rat brains under baseline and blocking (PF-04822163, 1 mg/kg) conditions, (b) TACs (0–60 min) of [^11^C]­PF-04822163 in brain regions of interest, and (c) area under the time-activity curve (0–20 min) of [^11^C]­PF-04822163 in rat brain regions of interest under baseline and blocking conditions. Data are means ± SD (*n* = 3) for the baseline study and means ± SEM (*n* = 2) for the blocking study. Co: cortex, St: striatum, Hi: hippocampus, Ce: cerebellum, and Po: pons.

To investigate the distribution of [^11^C]­PF-04822163 in the whole body, we conducted a biodistribution study in ddY mice (Figure 5). The radioactivity levels in major organs were assessed at 1, 5, 15, 30, and 60 min after the administration of [^11^C]­PF-04822163. Initially (at 1 and 5 min), a relatively high uptake (equal to or above 3% ID/g) was observed in the heart, lung, liver, kidney, adrenal, and brain. After the initial uptake, radioactivity accumulations decreased in almost all organs, while the uptake in the small intestine increased, and liver uptake remained at a high level (5% ID/g at 60 min), indicating a hepatobiliary elimination route. The brain uptake exhibited rapid washout after the initial high uptake, consistent with the results of PET imaging studies.

**Figure 5:**
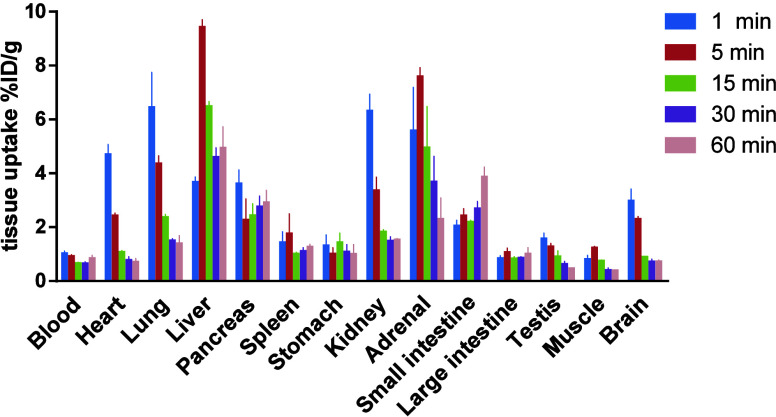
Whole-body biodistribution study of [^11^C]­PF-04822163 in ddY mice. All data are means ± SD, *n* = 3.

To gain insight into the *in vivo* stability of [^11^C]­PF-04822163, we performed radiometabolic analysis in the brain and plasma of SD rats (Figure 6). At 15 min postadministration of [^11^C]­PF-04822163, 95% of [^11^C]­PF-04822163 remained unchanged in the brain, while in plasma, 26% remained unchanged. The reasonable stability of [^11^C]­PF-04822163 in the brain indicated that the observed radioactivity accumulation in the brain predominantly originated from [^11^C]­PF-04822163.

**Figure 6:**
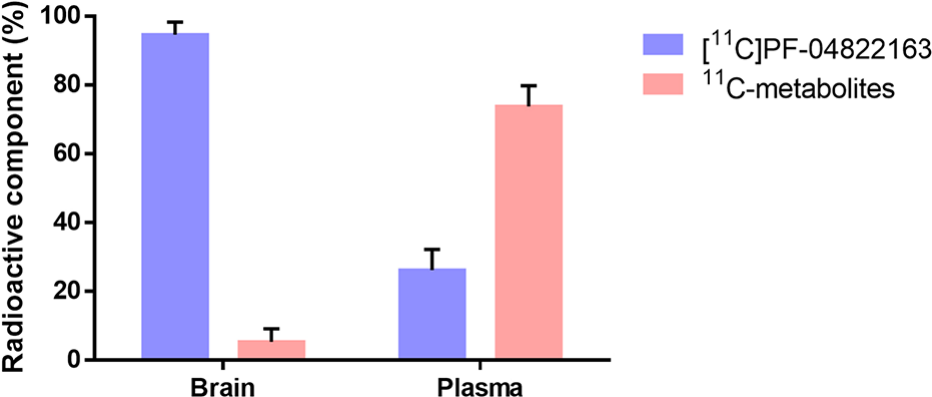
Radiometabolic analysis of [^11^C]­PF-04822163 in the brain and plasma of SD rats at 15 min. All data are means ± SD, *n* ≥ 2.

## Conclusions

We have developed [^11^C]­PF-04822163 as a potential PDE1 PET ligand. PF-04822163 exhibited excellent potency toward PDE1 and target selectivity in pharmacological evaluations *in vitro*. The PET ligand [^11^C]­PF-04822163 was labeled with favorable radiochemical yields (25 ± 10%, decay-corrected) and high molar activities (106–194 GBq/μmol). Further, *in vitro* and *in vivo* evaluations revealed that [^11^C]­PF-04822163 displayed marginal specific binding to PDE1 and adequate brain permeability. While [^11^C]­PF-04822163 demonstrated reasonable specific binding in autoradiography studies and exhibited stability in the brain and plasma, PET studies revealed efficient penetration but low retention in the rodent brain *in vivo*. These findings prompted us to focus on enhancing the pharmacological properties and increasing the binding affinity of our next-generation compounds through future structure–activity relationship (SAR) modifications. Furthermore, continuous efforts are essential to evaluate [^11^C]­PF-04822163 in higher species.

## Experimental Section

### General Information

Chemicals were used directly in synthesis as purchased. The NMR data were recorded by a 400 MHz NMR spectrometer. Coupling constants and chemical shifts were recorded in Hz and ppm, respectively. High-resolution mass data were collected in the APCI or ESI mode. All animal studies were approved by the Institutional Committee of the Massachusetts General Hospital (approval number 2020N000001). Rodents were fed under the 12h light/12h dark cycle.

#### Chemistry

##### Synthesis of 4-Formyl-2-methoxy-3-nitrophenyl Acetate (**2**)

4-Formyl-2-methoxyphenyl acetate **1** (75.0 g, 0.39 mol) was added to the fuming HNO_3_ (250 mL) at 5 °C and then stirred at 5 °C for 1 h. The reaction mixture was poured into ice-H_2_O, stirred for 30 min, and filtered. The solid was washed with H_2_O and dissolved in ethyl acetate, then washed with brine, dried over Na_2_SO_4_, and concentrated. The crude was washed with petroleum ether/methyl *t*-butyl ether (v/v = 2/1) to give 4-formyl-2-methoxy-3-nitrophenyl acetate **2** (68 g, 73%) as a yellow solid. ^1^H NMR (400 MHz, chloroform-*d*): δ 9.90 (s, 1H), 7.71 (d, *J* = 8.4 Hz, 1H), 7.44 (d, *J* = 8.4 Hz, 1H), 3.95 (s, 3H), 2.41 (s, 3H).

##### Synthesis of 4-Hydroxy-3-methoxy-2-nitrobenzoic Acid (**3**)

To a solution of 4-formyl-2-methoxy-3-nitrophenyl acetate **2** (50 g, 0.21 mol) in acetone (600 mL) was added KMnO_4_ (50 g, 0.316 mol) at 0 °C and then stirred at room temperature for 12 h. The reaction mixture was quenched by Na_2_SO_3_ (sat. aq.) and stirred for 10 min. Then, HCl (2 N) was added, and the resulting mixture was extracted with EtOAc. The organic phase was washed with brine, dried over Na_2_SO_4_, and concentrated to give 4-hydroxy-3-methoxy-2-nitrobenzoic acid **3** (white solid, 44 g, 98% yield) as an intermediate and used directly in the next step.

##### Synthesis of 2-Amino-4-hydroxy-3-methoxybenzoic Acid (**4**)

To a mixture of 4-hydroxy-3-methoxy-2-nitrobenzoic acid **3** (46 g, 0.216 mol) in MeOH (400 mL) was added Pd/C (10%, 4.6 g) and then stirred with H_2_ at room temperature for 12 h. The reaction mixture was filtered and concentrated to give 2-amino-4-hydroxy-3-methoxybenzoic acid **4** (brown solid, 39.5 g, 100%) as an intermediate and used directly in the next step.

##### Synthesis of 8-Methoxyquinazoline-4,7-diol (**5**)

To 2-amino-4-hydroxy-3-methoxybenzoic acid **4** (34 g, 0.186 mol) in 2-methoxyethanol (400 mL) was added formamidine acetate (34 g, 0.327 mol) and then stirred at 125 °C for 18 h. The reaction mixture was concentrated and purified by flash chromatography to give the crude. The crude was washed with petroleum ether/EtOAc (v/v = 1/3) to give 8-methoxyquinazoline-4,7-diol **5** (6.0 g, 17%) as a light brown solid. ^1^H NMR (400 MHz, DMSO-*d*_6_): δ 12.00 (s, 1H), 10.12 (s, 1H), 8.01 (s, 1H), 7.72 (d, *J* = 8.8 Hz, 1H), 7.04 (d, *J* = 8.8 Hz, 1H), 3.86 (s, 3H). Low-resolution mass spectra (LRMS, ESI, *m*/*z*): mass calcd for C_9_H_9_N_2_O_3_ (M + H^+^), 193.1; found, 193.2.

##### Synthesis of 4-Hydroxy-8-methoxyquinazolin-7-yl Acetate (**6**)

A mixture of 8-methoxyquinazoline-4,7-diol **5** (2.5 g, 13.0 mmol) in pyridine (400 mL) and Ac_2_O (2.1 g, 20.6 mmol) was stirred at 100 °C for 4 h. The reaction mixture was concentrated, and a mixed solvent of CH_2_Cl_2_/MeOH (5 mL/20 mL) was added. The resulting mixture was stirred for 1 h and concentrated. The residue was washed with petroleum ether to give 4-hydroxy-8-methoxyquinazolin-7-yl acetate **6** (brown solid, 2.6 g, 85%) as an intermediate and used directly in the next step.

##### Synthesis of 4-Chloro-8-methoxyquinazolin-7-yl Acetate (**7**)

Dry DMF (40 g, 0.55 mol) was added with SOCl_2_ (68.4 g, 0.57 mol) at 0 °C and then stirred at 40 °C for 2 h. The reaction mixture was concentrated and washed with petroleum ether to give the Vilsmeier reagent (70 g, 100%) a light-yellow solid. A solution of 4-hydroxy-8-methoxyquinazolin-7-yl acetate **6** (1.7 g, 7.26 mmol) in CH_2_Cl_2_ (40 mL) was added with the Vilsmeier reagent (26 g, 200 mmol) and then stirred at room temperature for 2 h. The reaction mixture was added with EtOAc, washed with NaHCO_3_ (sat. aq.), washed with brine, dried over Na_2_SO_4_, evaporated and purified by flash chromatography to give 4-chloro-8-methoxyquinazolin-7-yl acetate **7** (white solid, 1.0 g, 55%) as an intermediate, and used directly in the next step.

##### Synthesis of Methyl 1-(7-Acetoxy-8-methoxyquinazolin-4-yl)-6-chloro-2,3-dihydro-1*H*-indene-1-carboxylate (**8**)

A mixture of 4-chloro-8-methoxyquinazolin-7-yl acetate **7** (700 mg, 2.78 mmol) and methyl 6-chloro-2,3-dihydro-1*H*-indene-1-carboxylate (700 mg, 3.33 mmol) in THF (45 mL) was added with KHMDS (1 M in THF, 3.5 mL, 3.5 mmol) at 0 °C and stirred at 0 °C for 30 min. The reaction mixture was quenched with NH_4_Cl (sat. aq.) and extracted with EtOAc. The organic phase was washed with brine, dried over Na_2_SO_4_, and concentrated to give methyl 1-(7-acetoxy-8-methoxyquinazolin-4-yl)-6-chloro-2,3-dihydro-1*H*-indene-1-carboxylate **8** (yellow oil, 1.9 g, 84%, crude) as an intermediate and used directly in the next step.

##### Synthesis of 4-(6-Chloro-2,3-dihydro-1*H*-inden-1-yl)-8-methoxyquinazolin-7-ol (**9**)

A mixture of methyl 1-(7-acetoxy-8-methoxyquinazolin-4-yl)-6-chloro-2,3-dihydro-1*H*-indene-1-carboxylate **8** (1.9 g, crude) in NaOH (aq., 1 N, 25 mL)/MeCN (10 mL) was stirred at 130 °C (microwave) for 15 min. The reaction mixture was diluted with EtOAc, washed with citric acid (sat. aq.), H_2_O, and brine, dried over Na_2_SO_4_, and concentrated. The residue was purified by chromatography to give 4-(6-chloro-2,3-dihydro-1*H*-inden-1-yl)-8-methoxyquinazolin-7-ol **9** (yellow foam, 550 mg, 61% for 2 steps). ^1^H NMR (400 MHz, chloroform-*d*): δ 9.16 (s, 1H), 7.93 (d, *J* = 9.2 Hz, 1H), 7.39 (d, *J* = 9.2 Hz, 1H), 7.24 (d, *J* = 1.2 Hz, 1H), 7.18 (ddd, *J* = 8.0, 1.6, 0.8 Hz, 1H), 6.87 (t, *J* = 1.6 Hz, 1H), 6.60 (s, 1H), 5.26 (t, *J* = 8.0 Hz, 1H), 4.26 (s, 3H), 3.24 (dt, *J* = 16.0, 6.8 Hz, 1H), 3.07 (dt, *J* = 15.6, 8.0 Hz, 1H), 2.69–2.62 (m, 2H). LRMS (ESI, *m*/*z*): mass calcd for C_18_H_16_ClN_2_O_2_ (M + H^+^), 327.1; found, 327.1.

##### Synthesis of (*S*)-4-(6-Chloro-2,3-dihydro-1*H*-inden-1-yl)-8-methoxyquinazolin-7-ol (**10**)

The crude 4-(6-chloro-2,3-dihydro-1*H*-inden-1-yl)-8-methoxyquinazolin-7-ol **9** (550 mg, 1.69 mmol) was purified by a chiral column to give (*S*)-4-(6-chloro-2,3-dihydro-1*H*-inden-1-yl)-8-methoxyquinazolin-7-ol **10** (260 mg, 47%, 96% ee) as a yellow foam. HPLC purity: 98%. Melting point: 201–203 °C. ^1^H NMR (400 MHz, chloroform-*d*): δ 9.16 (s, 1H), 7.93 (d, *J* = 9.2 Hz, 1H), 7.39 (d, *J* = 8.8 Hz, 1H), 7.24 (s, 1H), 7.18 (ddd, *J* = 8.0, 2.0, 0.8 Hz, 1H), 6.87 (d, *J* = 1.6 Hz, 1H), 6.55 (s, 1H), 5.25 (t, *J* = 8.0 Hz, 1H), 4.27 (s, 3H), 3.23 (dt, *J* = 16.0, 6.4 Hz, 1H), 3.07 (dt, *J* = 16.4, 8.4 Hz, 1H), 2.68–2.62 (m, 2H). ^13^C NMR (100 MHz, chloroform-*d*): δ 171.7, 154.4, 152.2, 146.1, 145.1, 142.9, 139.3, 132.1, 127.5, 125.9, 124.9, 121.2, 119.7, 119.0, 62.5, 48.6, 33.2, 31.8. MS (ESI, *m*/*z*): 327.1 (M+ H^+^). HRMS (APCI): exact mass calcd for C_18_H_16_ClN_2_O_2_ (M + H^+^), 327.0895; found, 327.0898.

##### Synthesis of (*S*)-4-(6-Chloro-2,3-dihydro-1*H*-inden-1-yl)-7,8-dimethoxyquinazoline (PF-04822163)

To a mixture of **10** (20 mg, 0.06 mmol) and iodomethane (13 mg, 0.09 mmol) in MeCN (5 mL), K_2_CO_3_ (17 mg, 0.12 mmol) was added, and the mixture was heated at 40 °C and stirred for 1 h. After the reaction was complete, the reaction mixture was diluted with EtOAc and washed with H_2_O and brine, dried over Na_2_SO_4_, and concentrated. The residue was purified by prep-TLC to give PF-04822163 (20 mg, 90%) as a white solid. Melting point: 122–124 °C. [*a*]^20^_D_ = −0.073° (*c* = 2 mg/mL, CHCl_3_). ^1^H NMR (400 MHz, chloroform-*d*): δ 9.20 (s, 1H), 7.99 (d, *J* = 9.4 Hz, 1H), 7.44 (d, *J* = 9.4 Hz, 1H), 7.26 (d, *J* = 8.0 Hz, 1H), 7.18 (d, *J* = 8.4 Hz, 1H), 6.86 (s, 1H), 5.26 (t, *J* = 8.4 Hz, 1H), 4.15 (s, 3H), 4.09 (s, 3H), 3.28–3.03 (m, 2H), 2.67 (dd, *J* = 15.2, 7.6 Hz, 2H). ^13^C NMR (100 MHz, chloroform-*d*): δ 171.7, 154.9, 154.7, 146.2, 145.9, 142.9, 142.7, 132.1, 127.4, 125.9, 124.9, 120.8, 119.8, 115.6, 62.0, 56.8, 48.7, 33.1, 31.8. MS (ESI, *m*/*z*): 341.0 (M+ H^+^). HRMS (ESI): exact mass calcd for C_19_H_18_ClN_2_O_2_ (M + H^+^), 341.1051; found, 341.1053.

### Radiochemistry

[^11^C]­CH_3_I was prepared from [^11^C]­CO_2_ in a cyclotron and then transferred into a precooled reaction vial (−15 to −20 °C) containing the precursor **10** (1 mg), NaOH (0.5 M, 6.1 μL), and DMF (300 μL). After the transfer, this reaction mixture was heated at 30 °C for 5 min. Then, the HPLC mobile phase (CH_3_CN/H_2_O = 60/40, 0.5 mL) was added and then injected into HPLC for purification (column: CAPCELL PAK UG80 C18 column, 10 mm ID × 250 mm; mobile phase: CH_3_CN/H_2_O (60/40); flow rate: 5.0 mL/min). [^11^C]­PF-04822163 was obtained in 25 ± 10% radiochemical yield (decay-corrected, *n* = 5) based on [^11^C]­CO_2_. In addition, [^11^C]­PF-04822163 was generated with excellent radiochemical purity (>99%) and molar activity (106–194 GBq/μmol).

### Measurement of Log *D*

The measurement of log *D* was performed as previously reported.^30^ In brief, [^11^C]­PF-04822163, *n*-octanol (3 mL), and PBS (1×, 3 mL) were added to a centrifuge tube, and vortexing was conducted for 3 min followed by centrifugation (∼14,000 rpm) for 5 min. PBS (around 500 μL) and *n*-octanol (around 50 μL) were then weighted and measured by a gamma counter.

### *In Vitro* Autoradiography Studies

Autoradiography studies were performed according to previous reports.^30,31^ In brief, the rat brain sections (20 μm) were preincubated with a Tris–HCl buffer (50 mM) at room temperature for 20 min followed by incubation with [^11^C]­PF-04822163 (168 Bq/mL). For blocking studies, compound PF-04822163 (10 μM) or PF-04827736 (10 μM) was added to the incubation solution. Then, the brain sections were washed with ice-cold water thrice for 2 min and dipped in cold distilled water for 10 s. Subsequently, the brain slices were dried and positioned on imaging screens (BAS-MS2025, GE Healthcare, USA). Then, the screen was scanned by an Amersham Typhoon 5 analyzer system. The radioactivity values were normalized as a percentage of the radioactivity relative to the control.

### PET Imaging Studies in Rat Brains

PET imaging was conducted as previously reported.^30,31^ An Inveon PET scanner (Siemens) was used for dynamic PET imaging studies, [^11^C]­PF-04822163 (*ca*. 17.5 MBq/1.5 mL) was intravenously injected via the tail vein via a preinstalled catheter, and dynamic PET images were acquired in a 3D mode for a period of 60 min. In blocking studies, PF-04822163 (1 mg/kg) was injected 10 min before administration of [^11^C]­PF-04822163. The dynamic PET images were reconstructed using ASIPro VW software, and volumes of interest were calculated.

### Whole-Body Biodistribution Study in Mice

The biodistribution experiment was performed according to previous reports.^30,31^ In brief, [^11^C]­PF-04822163 (4.44 MBq/0.1 mL) was injected (i.v.) into ddY mice via the tail vein. After the administration of [^11^C]­PF-04822163, the mice were sacrificed at different time points (1, 5, 15, 30, and 60 min), and tissues of interest were collected. The tissues were weighted and measured by a gamma counter.

### Radiometabolic Analysis in the Rat Brain and Plasma

The radiometabolic analysis was performed following a previous report.^30^ After the intravenous injection of [^11^C]­PF-04822163, SD rats were sacrificed at 15 min postinjection. Plasma samples were quenched with acetonitrile and centrifuged, and the supernatant was assessed by radio-HPLC. The brain was collected and homogenized and quenched with acetonitrile, and the resulting homogenate was centrifuged. The supernatant was analyzed by radio-HPLC.

## Supplementary Material



## Notes

Detailed materials and methods and additional figures and tables (PDF)
