# Supplementary material for: Development of a Candidate 11C‑Labeled Selective Phosphodiesterase 1 Radioligand for Positron Emission Tomography
Source: ACS Omega. 2024 Oct 21;9(44):44154–63. doi: 10.1021/acsomega.4c03214 (PMC11541501; doi:10.1021/acsomega.4c03214)

Supporting Information

Development of a Candidate ^{11}C-Labeled Selective Phosphodiesterase 1 Radioligand for Positron Emission Tomography

Jian Rong[1,2,†], Tomoteru Yamasaki[3,†], Jiahui Chen[1,2], Katsushi Kumata[3], Chunyu Zhao[1,2], Masayuki Fujinaga[3], Kuan Hu[3], Wakana Mori[3], Yiding Zhang[3], Lin Xie[3], Ahmad F. Chaudhary[1], Xin Zhou[1], Wei Zhang[1], Yabiao Gao[1], KuoZhang[1], Jimmy S. Patel[1,,4], Zhendong Song[1], Thomas L. Collier[1,2], Hongjie Yuan[5], Chongzhao Ran[6], Achi Haider[1,2], Yinlong Li[1,2], Ming-Rong Zhang[3,*], and Steven Liang[1,2,*]

[1]Department of Radiology and Imaging Sciences, Emory University, Atlanta, GA 30322, USA

[2]Division of Nuclear Medicine and Molecular Imaging, Massachusetts General Hospital & Department of Radiology, Harvard Medical School, Boston, MA 02114, USA

[3]Department of Advanced Nuclear Medicine Sciences, Institute for Quantum Medical Science, National Institutes for Quantum Science and Technology, Chiba, Chiba, 263-8555, Japan

[4]Department of Radiation Oncology, Winship Cancer Institute of Emory University, Atlanta, GA 30322, USA.

[5]Department of Pharmacology and Chemical Biology, Emory University School of Medicine, Atlanta, GA 30322, USA.

[6]Athinoula A. Martinos Center for Biomedical Imaging, Department of Radiology, Massachusetts General Hospital and Harvard Medical School, Boston, MA 02114, USA.

*Correspondence: zhang.ming-rong@qst.go.jp, steven.liang@emory.edu

†These authors contributed equally to this work.

Table of contents

Supporting Figures and Table 3

Figure S1 Representative PDE1 inhibitors. 3

Figure S2 Off-target pharmacological evaluation of PF-04822163 4

Figure S3 Area under time-activity-curve of PET studies with [^{11}C]PF-04822163 in brain regions of interest under baseline and blocking conditions. 4

Table S1 Whole-body biodistribution study of [^{11}C]PF-04822163 in ddY mice. 5

Supercritical fluid chromatograms (SFC)...5

HPLC radio-chromatograms 7

^{1}H NMR and ^{13}C NMR spectra 11

1 Supporting Figures and Table

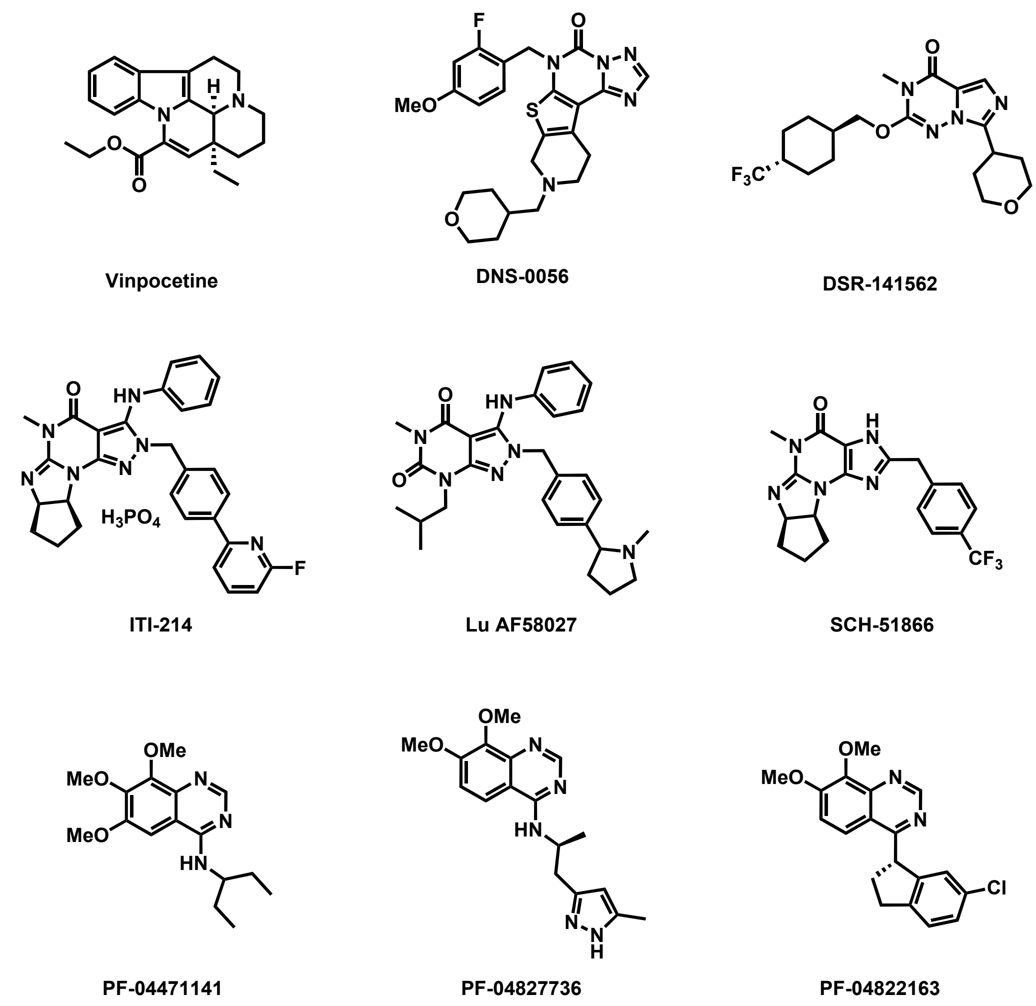

Figure S1. Representative PDE1 inhibitors.

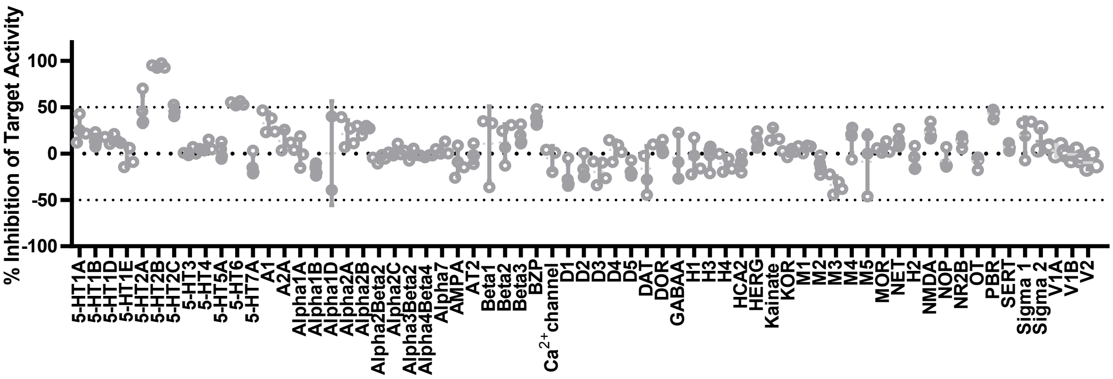

Figure S2. Off-target pharmacological evaluation of compound PF-04822163 against major CNS targets, including common GPCRs, enzymes, ion channels, and transporters: initial screening at a concentration of 10 μM (supported by the NIMH PDSP). All data are mean ± SD ($n \geq 3$). No significant off-target binding (> 50%) was observed at 10 μM compound testing concentration, except 5-HT2B (Ki = 262 nM) and 5-HT6 (Ki = 4858 nM).

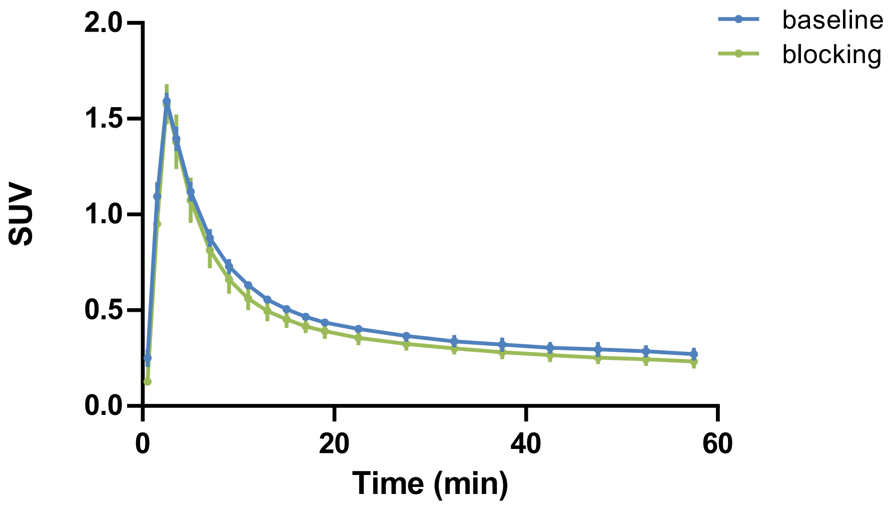

Figure S3. The time-activity-curves (0-60 min) of PET studies with [^{11}C]PF-04822163 in rat brains under baseline and blocking conditions. All data were referred to as mean ± SEM, $n \geq 2$.

Table S1. Whole-body ex vivo biodistribution study of [^{11}C]PF-04822163 in ddY mice.

Raw data

%ID/g	1 min			5 min			15 min			30 min			60 min		
Blood	0.989	0.968	1.122	0.984	0.875	0.893	0.658	0.694	0.633	0.579	0.709	0.663	0.973	0.778	0.791
Heart	4.512	4.47	5.123	2.421	2.335	2.528	1.021	1.118	1.098	0.671	0.914	0.763	0.854	0.635	0.659
Lung	5.926	5.509	7.927	4.205	4.205	4.685	2.255	2.428	2.434	1.455	1.572	1.497	1.729	1.228	1.225
Liver	3.475	3.815	3.749	9.683	9.126	9.467	6.359	6.69	6.409	4.226	4.695	4.895	5.84	4.629	4.367
Pancreas	3.236	3.441	4.19	2.857	2.559	1.388	2.32	2.917	2.076	2.532	3.215	2.552	2.608	2.71	3.441
Spleen	1.226	1.892	1.184	1.455	1.234	2.596	0.948	1.054	1.031	1.25	0.99	1.089	1.285	1.357	1.181
Stomach	1.751	1.228	0.973	1.271	0.852	0.904	1.783	1.421	1.101	0.965	1.394	0.892	1.396	0.895	0.73
Kidney	5.657	6.446	6.864	3.8	2.839	3.453	1.903	1.838	1.758	1.463	1.372	1.656	1.502	1.582	1.526
Adrenal	6.724	6.287	3.76	7.436	7.974	7.367	6.689	3.805	4.356	3.15	4.778	3.128	1.928	3.208	1.784
Small intestine	1.918	2.282	1.969	2.635	2.142	2.51	2.213	2.243	2.125	2.427	2.94	2.714	4.277	3.753	3.584
Large intestine	0.749	0.875	0.918	1.146	0.888	1.169	0.793	0.918	0.81	0.857	0.908	0.813	1.26	0.955	0.822
Testis	1.799	1.537	1.404	1.412	1.225	1.211	1.123	0.699	0.906	0.615	0.548	0.736	0.464	0.478	0.474
Muscle	0.694	0.966	0.788	1.272	1.26	1.179	0.751	0.777	0.736	0.315	0.449	0.458	0.405	0.389	0.386
Brain	2.571	2.933	3.434	2.286	2.214	2.404	0.898	0.913	0.881	0.65	0.826	0.694	0.784	0.691	0.72

Data are mean ± SD, n = 3

	1 min			5 min			15 min			30 min			60 min		
%ID/g	Mean	SD	N	Mean	SD	N	Mean	SD	N	Mean	SD	N	Mean	SD	N
Blood	1.026	0.083	3	0.917	0.058	3	0.662	0.031	3	0.650	0.066	3	0.847	0.109	3
Heart	4.701	0.365	3	2.428	0.097	3	1.079	0.052	3	0.783	0.123	3	0.716	0.120	3
Lung	6.454	1.293	3	4.365	0.277	3	2.372	0.102	3	1.508	0.059	3	1.394	0.290	3
Liver	3.680	0.180	3	9.425	0.281	3	6.486	0.178	3	4.605	0.343	3	4.945	0.786	3
Pancreas	3.622	0.503	3	2.268	0.777	3	2.437	0.433	3	2.766	0.389	3	2.919	0.454	3
Spleen	1.434	0.397	3	1.762	0.731	3	1.011	0.056	3	1.110	0.131	3	1.274	0.088	3
Stomach	1.318	0.397	3	1.009	0.229	3	1.435	0.341	3	1.084	0.271	3	1.007	0.347	3
Kidney	6.322	0.613	3	3.364	0.487	3	1.833	0.072	3	1.497	0.145	3	1.537	0.041	3
Adrenals	5.590	1.600	3	7.592	0.332	3	4.950	1.531	3	3.685	0.946	3	2.307	0.784	3
Small intestine	2.056	0.197	3	2.429	0.256	3	2.194	0.061	3	2.694	0.257	3	3.871	0.361	3
Large intestine	0.848	0.088	3	1.067	0.156	3	0.840	0.068	3	0.859	0.048	3	1.012	0.225	3
Testis	1.580	0.201	3	1.282	0.112	3	0.909	0.212	3	0.633	0.095	3	0.472	0.007	3
Muscle	0.816	0.138	3	1.237	0.050	3	0.755	0.021	3	0.407	0.080	3	0.393	0.010	3
Brain	2.979	0.433	3	2.301	0.096	3	0.897	0.016	3	0.723	0.092	3	0.732	0.048	3

Supercritical fluid chromatograms (SFC) of compound 10 (final chiral precursor) and its (*R*)-isomer

Preparative separation method:

Instrument: MG Ⅱ preparative SFC (SFC-14)

Column: ChiralPak AD, 250 × 30 mm I.D., 5 μm

Mobile phase: A for CO_2 and B for Ethanol

Gradient: B 40%

Flow rate: 80 mL /min

Back pressure: 100 bar

Column temperature: 38 ℃

Wavelength: 220 nm

Analytical method:

Instrument: Waters UPC2 analytical SFC (SFC-H)

Column: ChiralPak AD, 150 × 4.6 mm I.D., 3 μm

Mobile phase: A for CO_2 and B for Ethanol (0.05% DEA)

Gradient: B 40%

Flow rate: 2.5 mL/min

Back pressure: 100 bar

Column temperature: 35 ℃

Wavelength: 220 nm

Supercritical fluid chromatogram (SFC) of the racemic sample

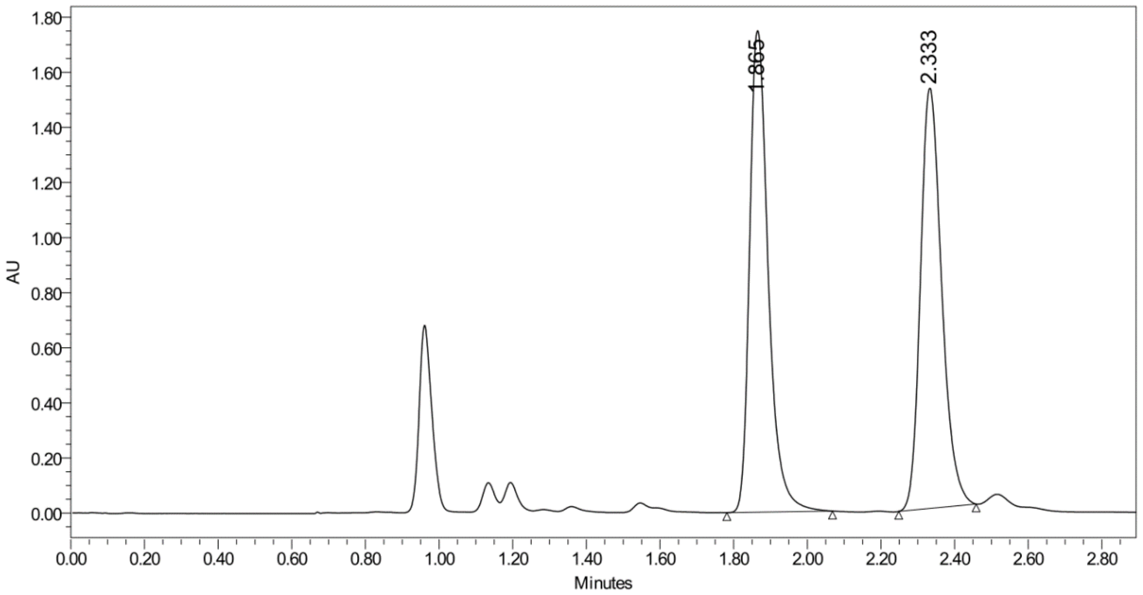

Supercritical fluid chromatogram (SFC) of (*R*)-isomer

(*R*)-isomer: t_R = 1.8 min (99.2% ee)

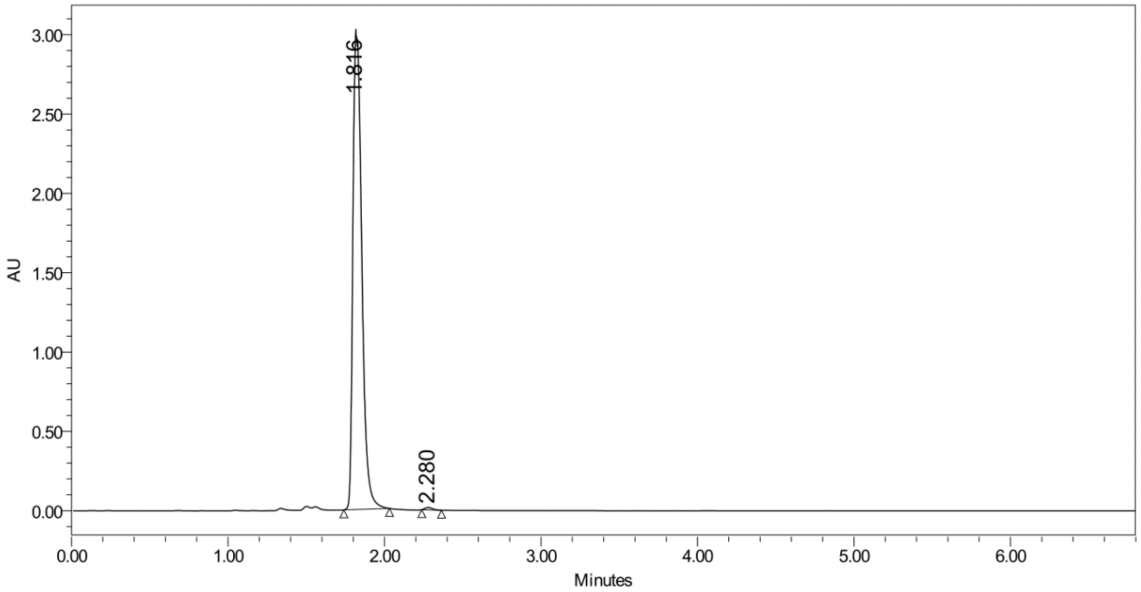

Supercritical fluid chromatogram (SFC) of compound **10** (final chiral precursor)

compound **10**: t_R = 2.3 min (98.1 % ee)

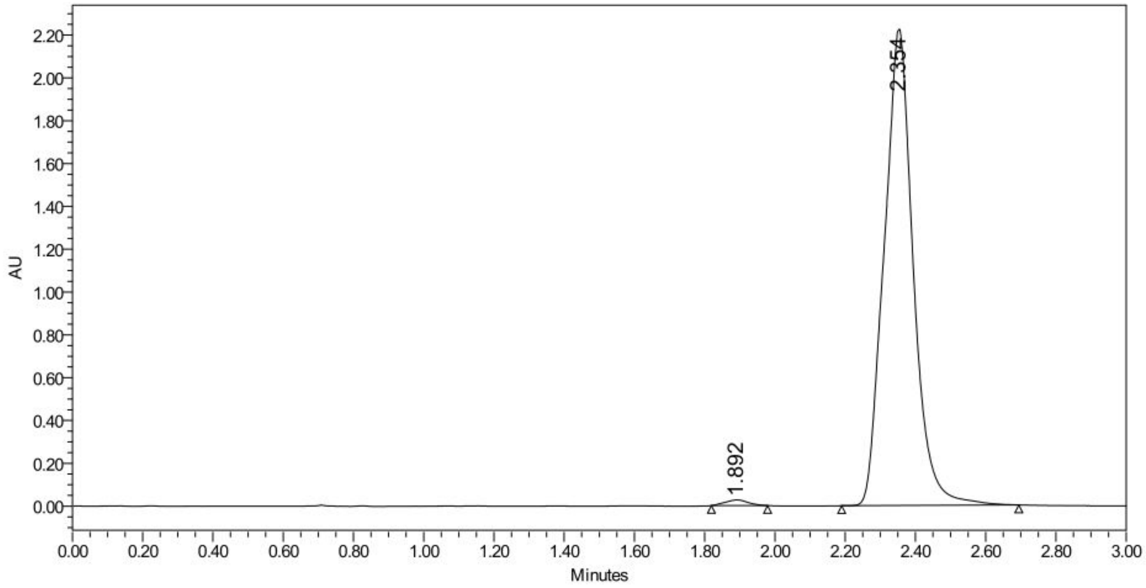

HPLC radio-chromatograms of [^{11}C]PF-04822163

Semi-prep radio-HPLC chromatogram of [^{11}C]PF-04822163

Column: CAPCELL PAK (10 ×250 mm)

Mobile phase: CH_3CN-H_2O=60%-40%

flow rate: 5.0 mL/min, UV: 254 nm

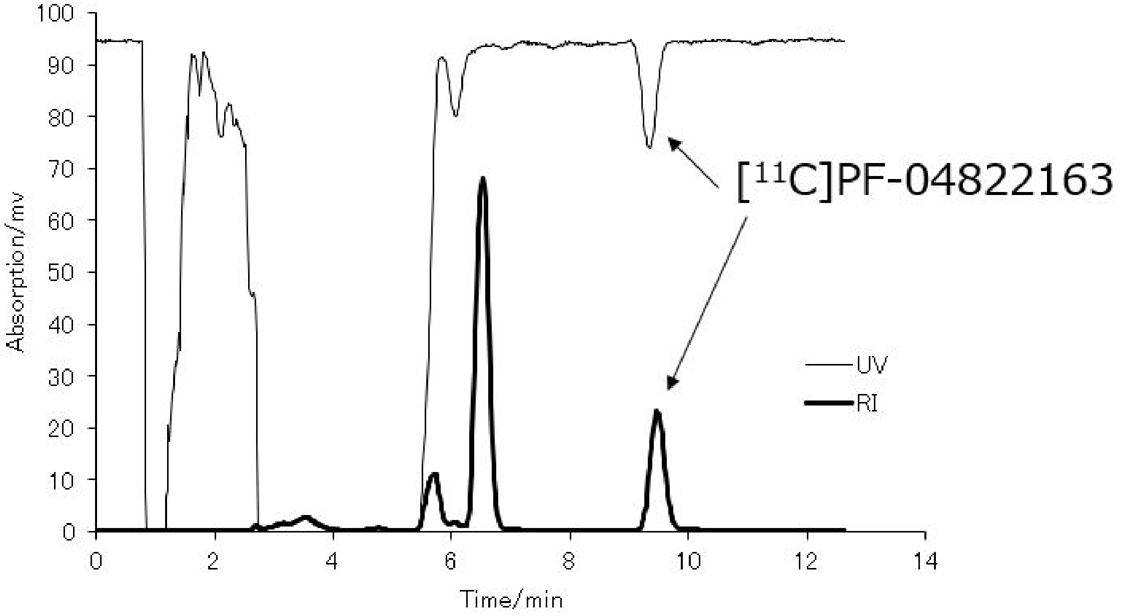

Analytical radio-HPLC chromatogram of [11C]PF-04822163

Chiral column: CHIRALPAK AS-RH (4.6 ×150 mm)

Mobile phase: CH_3CN-H_2O = 40%-60%

flow rate: 1.0 mL/min

UV: 254 nm

Purity: 98% (96% ee)

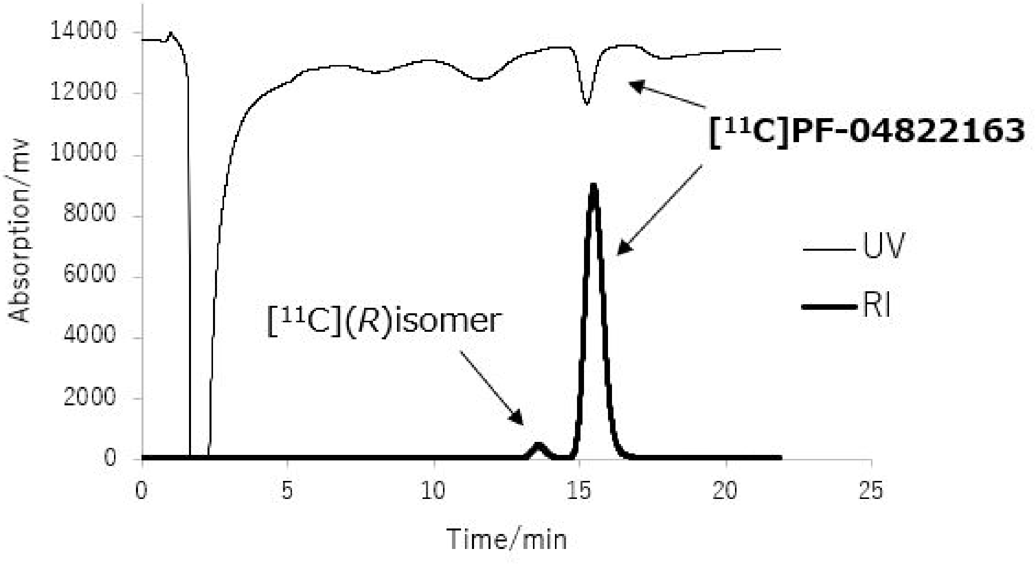

Co-injection of [^{11}C]PF-04822163 with unlabeled PF-04822163

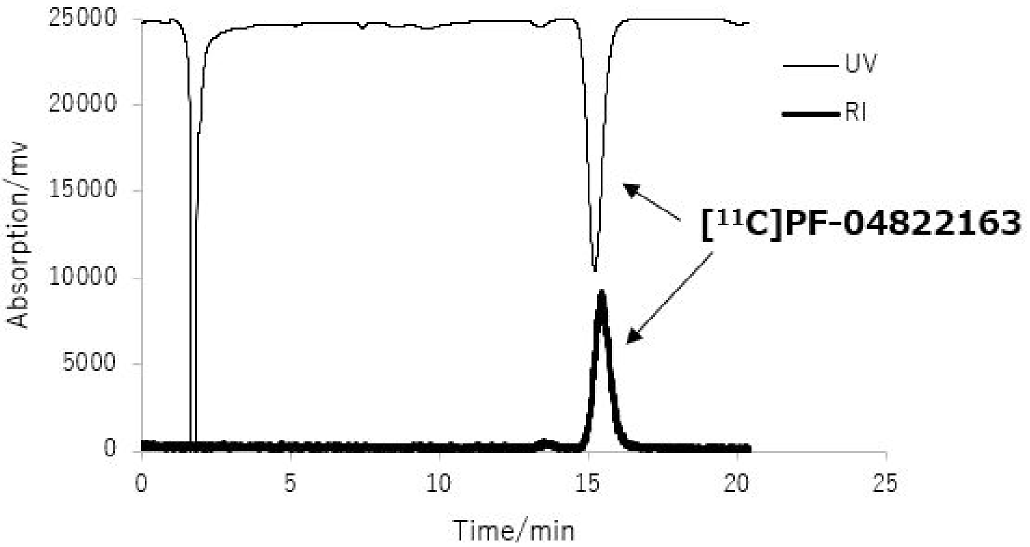

Calibration curve of compound PF-04822163

The specific activities of [^{11}C]PF-04822163 were calculated based on the calibration curve.

	1/100	1/10	undiluted solution
concentration (μmol/mL)	0.0001	0.0059	0.0587
area	1026	168729	1253904

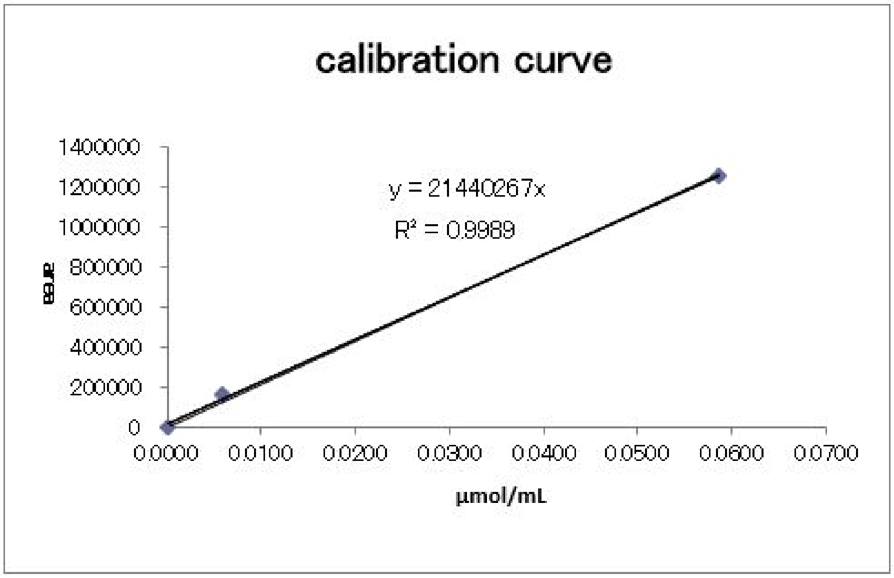

Radio-chromatograms of metabolite analysis

Radio-chromatograms of the metabolite analysis in the brain

At 15 minutes post-administration of [^{11}C]PF-04822163, 95% of [^{11}C]PF-04822163 remained unchanged in rat brains (n = 2).

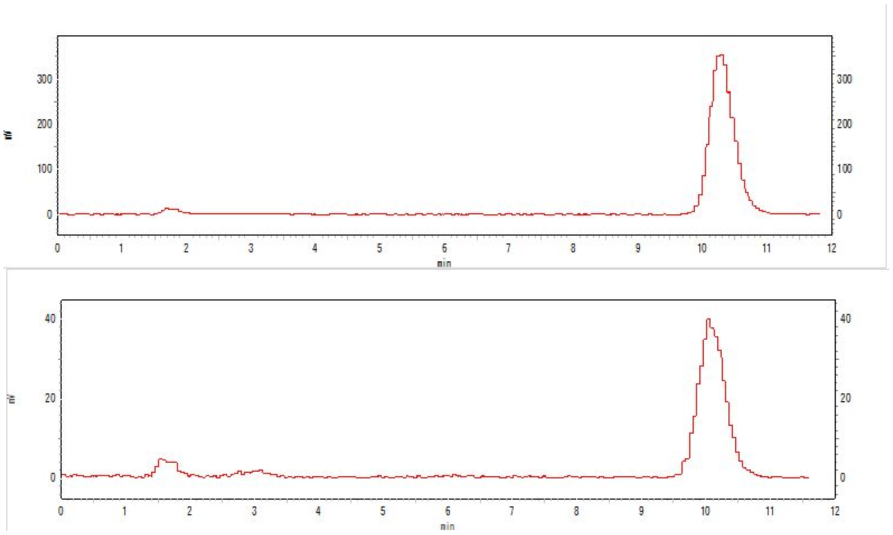

Radio-chromatograms of the metabolite analysis in plasma

At 15 minutes post-administration of [^{11}C]PF-04822163, 26% of [^{11}C]PF-04822163 remained unchanged in rat plasma (n = 2).

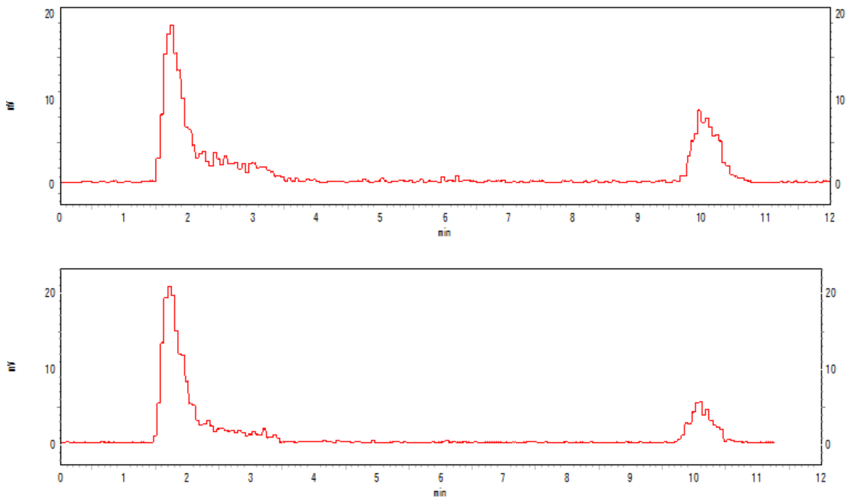

NMR spectra of compounds 10 and PF-04822163

^{1}H NMR and ^{13}C NMR spectra of compound **10** ($CDCl_3$, 400 MHz)

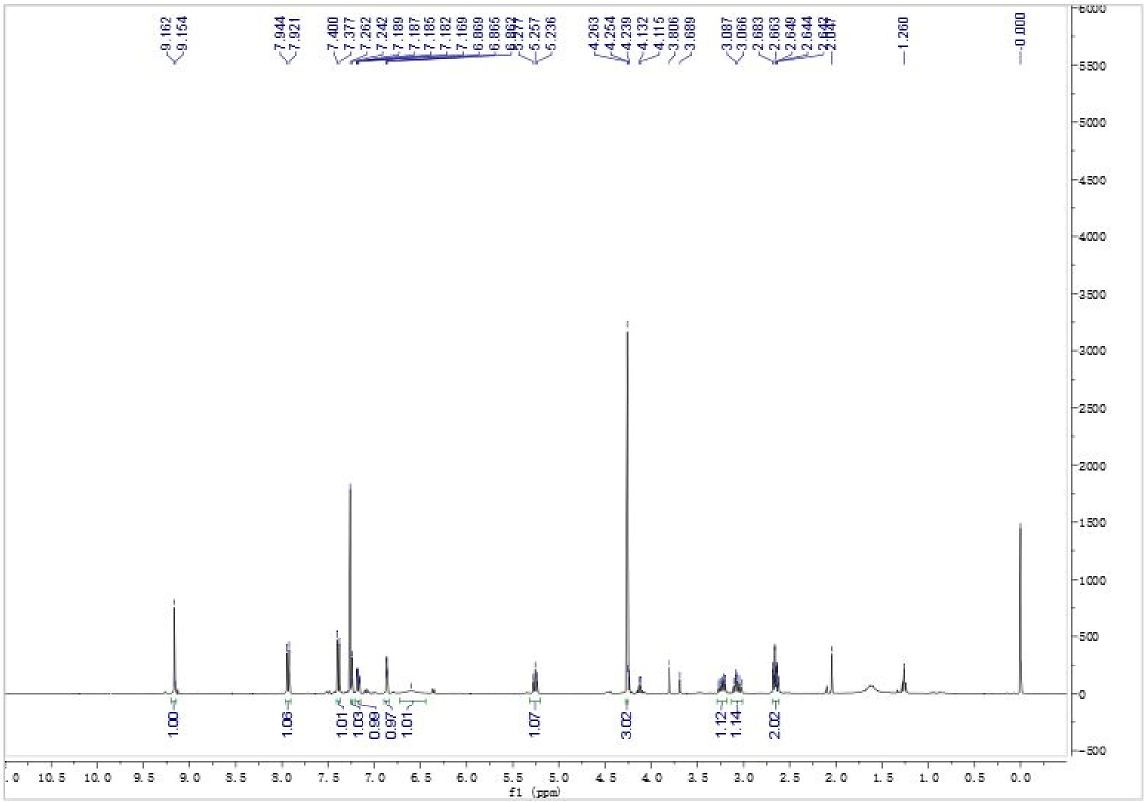

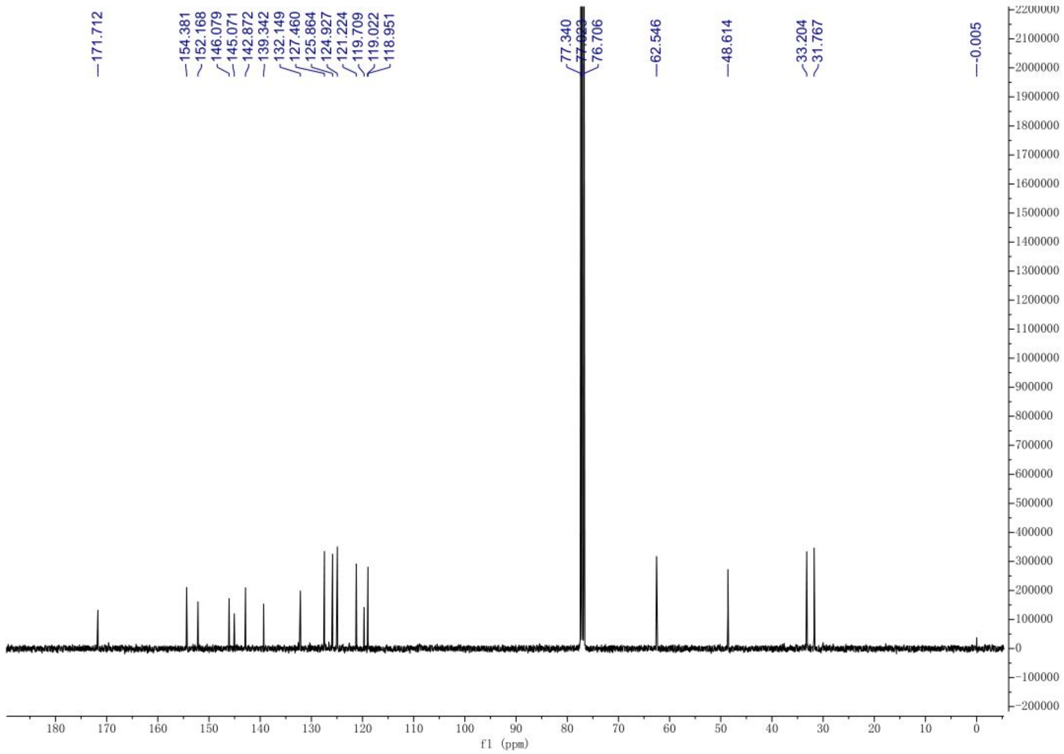

^{1}H NMR and ^{13}C NMR spectra of PF-04822163 ($CDCl_3$, 400 MHz)

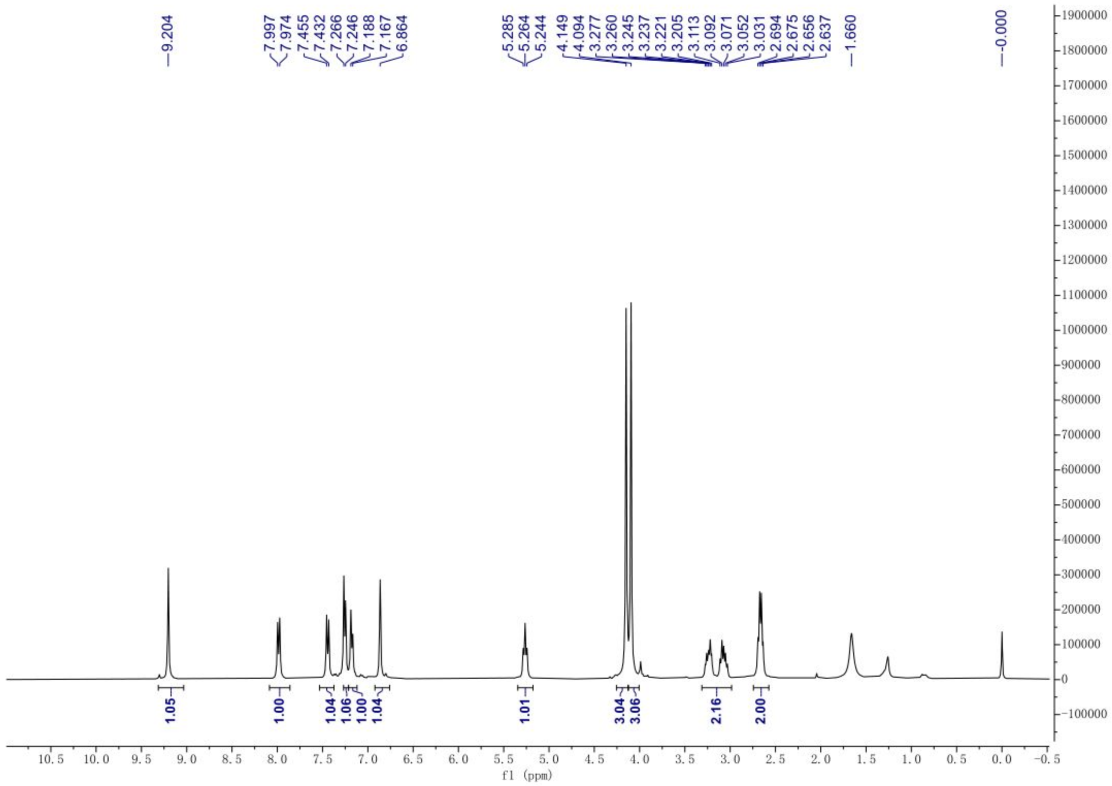

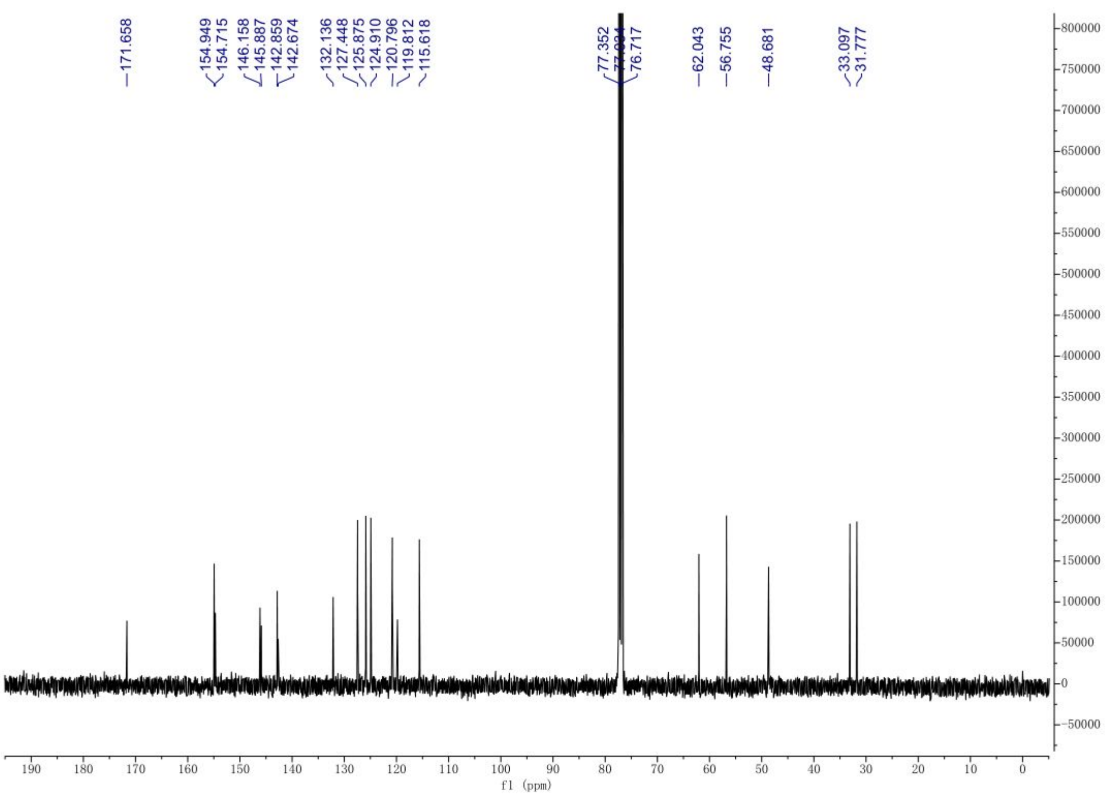

Supplement: Supplementary file 1 [file ao4c03214_si_001.pdf]
